# Domestic Summary

**Published:** 1838-11-07

**Authors:** 


### DOMESTIC SUMMARY.

*Medical Prize Questions.*—The questions for the Fiske Fund prize, for 1839, are,—First. The Medical Botany of Rhode Island. Second. Erysipelas, its varieties and the best mode of treatment. Dissertations to be sent, free of expense, before the first of May, to M. Parsons, M. D., Providence; N. Manchester, M. D., North Providence; or E. Fowler, M. D., Smithfield, Rhode Island. The Boylston prize questions, for 1839, are,—First. The Pathology and Treatment of Rheumatism. Second. What is Scrofula? and what its best mode of treatment? For 1840—First. The Pathology and Treatment of Typhus and Typhoid Fever. Second. The Pathology and Treatment of Medullary Sarcoma. The prize of the Boylston Essays is a gold medal, or fifty dollars. Dissertations for each year are to be transmitted before the first Wednesday of April, to John C. Warren, M. D., Boston, Massachusetts.

At the sixth annual meeting of the British Provincial Medical and Surgical Association, at the city of Bath, Dr. J. C. Warren, of Boston, and Dr. R. Dunglison, of Philadelphia, were appointed honorary corresponding members.

